# Loss of N-Cadherin Expression in Tumor Transplants Produced From As^+3^- and Cd^+2^-Transformed Human Urothelial (UROtsa) Cell Lines

**DOI:** 10.1371/journal.pone.0156310

**Published:** 2016-05-25

**Authors:** Elizabeth J. Sandquist, Seema Somji, Jane R. Dunlevy, Scott H. Garrett, Xu Dong Zhou, Andrea Slusser-Nore, Donald A. Sens

**Affiliations:** 1 Department of Pathology, School of Medicine and Health Sciences, University of North Dakota, Grand Forks, North Dakota, United States of America; 2 Department of Basic Sciences, School of Medicine and Health Sciences, University of North Dakota, Grand Forks, North Dakota, United States of America; University of Colorado School of Medicine, UNITED STATES

## Abstract

**Background:**

Epithelial to mesenchymal transition is a process in which a cell experiences a loss of epithelial cell characteristics and acquires a more mesenchymal cell phenotype. In cancer, epithelial to mesenchymal transition has been proposed to play an important role during specific stages of tumor progression. The role epithelial to mesenchymal transition and mesenchymal to epithelial transition might play in toxicant-induced urothelial cancer is unknown.

**Methods:**

Real-time PCR, Western blotting, immuno-histochemistry and immuno-fluorescence were used to determine the expression of E- and N-cadherin in the UROtsa parent, the As^+3^- and Cd^+2^-transformed cell lines, the spheroids isolated from these cell lines as well as the tumor heterotransplants that were produced by the injection of the transformed cells into immune compromised mice.

**Results:**

This study showed that N-cadherin expression was increased in 6 As^+3^- and 7 Cd^+2^- transformed cell lines generated from human urothelial cells (UROtsa). The expression varied within each cell line, with 10% to 95% of the cells expressing N-cadherin. Tumors produced from these cell lines showed no expression of the N-cadherin protein. Spheroids which are made up of putative cancer initiating cells produced from these cell lines showed only background expression of N-cadherin mRNA, increased expression of aldehyde dehydrogenase 1 mRNA and produced tumors which did not express N-cadherin. There was no change in the expression of E-cadherin in the tumors, and the tumors formed by all the As^+3^ and Cd^+2^-transformed cell lines and cancer initiating cells stained intensely and uniformly for E-cadherin.

**Conclusions:**

The finding that the cells expressing N-cadherin gave rise to tumors with no expression of N-cadherin is in agreement with the classical view of epithelial to mesenchymal transition. Epithelial to mesenchymal transition and N-cadherin are associated with dissemination and not with the ability to establish new tumor growth. Mesenchymal to epithelial transition and E-cadherin are viewed as necessary for a cell to establish a new metastatic site. The lack of N-cadherin expression in tumor transplants is consistent with E-cadherin expressing cells “seeding” a site for tumor growth. The study shows that a minority population of cultured cells can be the initiators of tumor growth.

## Introduction

The epithelial to mesenchymal transition (EMT) is defined as a process in which a cell experiences a loss of epithelial cell characteristics and acquires a more mesenchymal cell phenotype. In cancer, EMT has been proposed to play an important role during specific stages of tumor progression, such as invasion and intravasation, where tumor cells disassemble and migrate to tissue and/or organ sites distant from the primary tumor [1–3]; however evidence supporting a complete EMT of epithelial cells to mesenchymal cells is lacking [4]. In many instances, EMT in tumor progression is associated with a process called “cadherin switching.” Cadherin switching is based on the concept that in normal tissues, epithelial and mesenchymal cells mainly express E-cadherin and N-cadherin, respectively. However, in various types of malignant tumors originating from epithelial cells, it has been observed that the down-regulation of E-cadherin and/or up-regulation of N-cadherin is associated with the acquisition of aggressive tumor phenotypes [5, 6]. Cadherin switching and alterations in the expression of E-cadherin and N-cadherin have been documented to occur in urothelial cancer [7]. The loss of E-cadherin expression in bladder cancer is associated with increased stage, grade, and incidence of recurrence and with decreased survival rate [8–14]. Similarly, in several of the above studies, an increase in N-cadherin expression has been positively correlated with bladder cancer progression and recurrence [9–11]. In addition, a study of 181 bladder cancer patients stratified by tumor invasiveness found that N-cadherin expression negatively correlates to survival in all cases, but positively associates with survival in invasive cases [15]. No correlation was found between N-cadherin and stage, grade, lymph node involvement or vascular invasion in a study of 572 bladder cancer patients [8].

The development of urothelial cancer has a strong association with the environment [16]. This laboratory is interested in exploring the relationship between arsenite (As^+3^) and cadmium (Cd^+2^) exposure and the development of urothelial cancer. Exposure to environmental arsenic is strongly associated with urothelial cancer, and high incidences of this cancer are also linked to localities that exhibit increased rates of arsenic-induced skin cancer [17–22]. Studies involving arsenic exposed human subjects that developed urothelial carcinomas showed that these subjects expressed low levels of the miRNA family members that are known to be tumor suppressive and regulate the process of EMT [23]. This suggests that arsenic exposure may be able to induce a dedifferentiation program resulting in a more malignant type cancer. A recent study suggests that exposure of populations to150 μg/L of arsenic in drinking water increases their risk of dying from bladder cancer by 30% when compared to those exposed to10 μg/L. In addition, exposure to 10 μg/L of arsenic in the drinking water doubles the risk of the exposed population when compared to the unexposed population [24]. The high association of smoking with urothelial cancer may indirectly involve Cd^+2^ exposure due to the high accumulation of this metal seen in smokers [25, 26], and there are several epidemiological studies that implicate this metal in the development of urothelial cancer [27, 28]. In the Belgium case-control study on bladder cancer it was found that the odds ratios for bladder cancer occurrence with corresponding 95% confidence intervals was 8.3 in individuals that had elevated blood cadmium levels [27]. The human urothelial cell line, UROtsa, has been utilized by this laboratory as a model to investigate the relationship between As^+3^ and Cd^+2^ exposure and the development of urothelial cancer. The cell line, immortalized via a temperature-sensitive large T-antigen, is non-tumorigenic, retains features of transitional urothelium when propagated on serum -free growth medium and is thus, an excellent *in-vitro* model of human urothelium [29]. Using this cell line, it was shown that Cd^+2^ and As^+3^ can cause the malignant transformation of urothelial cells as evidence by colony formation in soft agar and tumor formation in athymic mice [30–32]. This system of metal-transformation was also used to develop multiple transformed isolates to study cancer heterogeneity [33]. These tumors exhibit morphological features consistent with human urotehlial cancer; the most prominent type of bladder cancers found in western countries [16]. The UROtsa cell line was used in the present study to determine if exposure to, or malignant transformation by, As^+3^ or Cd^+2^ would alter the expression of E- or N-cadherin *in*-*vitro* and *in*-*vivo*.

## Materials and Methods

### Animals

Mouse heterotransplants of the UROtsa transformed cell cultures were produced by subcutaneous injection at a dose of 1 X 10^6^ cells in the dorsal thoracic midline of athymic nude (NCR-*nu/nu*) mice. Tumor formation and growth were assessed externally with a ruler on a weekly basis and the maximal tumor size was 2.5 cm. All mice were sacrificed by 10 weeks after injection or when clinical conditions dictated euthanasia (excessive weight loss, lethargy, self-mutilation or mutilation by cage mates). This study adhered to all recommendation dictated in the Guide for the Care and Use of Laboratory Animals of the NIH. The specific protocol was approved by the University of North Dakota Animal Care Committee (IACUC#1110-2C). All efforts were taken in order to minimize animal suffering, and mice were euthanized when clinical condition dictated. Animals were sacrificed by 10 weeks post tumor transplantation by CO_2_ inhalation and euthanasia conformed to AVMA Guideline on Euthanasia.

### Cell culture

The UROtsa cell line was initially obtained from John Masters formerly of the Institute of Urology, University College London and maintained in 75 cm^2^ tissue culture flasks using Dulbecco’s modified Eagle’s medium (DMEM) containing 5% v/v fetal calf serum in a 37°C, 5% CO_2_: 95% air atmosphere [29]. The isolation and growth of the 7 isolates of the Cd^+2^- transformed lines and the 6 individual isolates of the As^+3^-transformed UROtsa cells has been described previously [30–32]. The transformed cell lines were all grown and maintained using identical conditions. Confluent flasks were sub-cultured at a 1:4 ratio using trypsin-EDTA (0.05%, 0.02%) and the cells were fed fresh growth medium every 3 days. For long term exposure to As^+3^ and Cd^+2^, the UROtsa parental cells were exposed to 1.0 μM NaAsO_2_ or CdCl_2_ and passaged in the presence of NaAsO_2_ or CdCl_2_ for 30 days. During this exposure period, the ability of the cells to form colonies in soft agar was determined every five passages using protocols described previously [30–32]. Cells were harvested for PCR and Western blot analysis at each passage. The J82, RT4 and TCCSUP human bladder cancer cell lines were obtained from the American Type Culture Collection (ATCC, Rockville, MD) and were grown in DMEM supplemented with 5% (v/v) fetal calf serum, fed every 3 days, and routinely passaged at a 1:4 ratio upon attaining confluence.

For the generation of spheroids, the As^+3^-and Cd^+2^-transformed cell lines were seeded at a density of 10^5^ cells in T-25 cm^2^ Ultra-low attachment flasks (Corning Inc., Corning NY). The growth medium consisted of a 1:1 mixture of Dulbecco’s modified Eagles’ medium and Hams’s F-12 growth medium supplemented with selenium (5 ng/ml), insulin (5 μg/ml), transferrin (5 μg/ml), hydrocortisone (36 ng/ml), triiodothyronine (4 pg/ml), and epidermal growth factor (10 ng/ml). The cells were allowed to form spheres for 8 days after which time the spheroids were harvested by centrifugation for RNA and protein isolation, and for injection into immune compromised mice. The protocol for the transplantation of the transformed UROtsa cell lines into immune compromised mice has been described previously [30].

### Expression of E- and N-cadherin mRNA and protein in the UROtsa derived cell lines and transplanted tumors

The preparation of total RNA and protein from cultured cells and tissues have been described previously [30–32]. The expression of E- and N-cadherin was determined using real-time RT-PCR and primers were obtained from Bio-Rad and Qiagen, respectively. Briefly, 1 μg of purified RNA was subjected to complementary DNA (cDNA) synthesis using the iScript cDNA synthesis kit (Bio-Rad Laboratories) in a total volume of 20 μL. Real-time RT-PCR was performed utilizing the SYBR Green kit (Bio-Rad Laboratories) with 2 μL of cDNA, 0.2 μM primers in a total volume of 20 μL in an iCycler iQ real-time detection system (Bio-Rad Laboratories). Amplification was monitored by SYBR Green fluorescence. The expression data was normalized to the expression of β-actin as described previously [34].

The expression of E- and N-cadherin protein was determined by Western blotting using protocols that have been previously published by this laboratory [30–32]. Briefly, total cellular protein (15 μg) was separated by SDS-PAGE using the TGX AnyKd SDS polyacrylamide gel (Bio-Rad Laboratories). Samples were reduced in β-mercaptoethanol and boiled for 5 min prior to loading. Protein was transferred to a 0.2 μm PVDF membrane followed by blocking in 5% nonfat dry milk in Tris buffered saline (TBS) supplemented with 1% Tween 20 (TBS-T) for 90 min. After blocking, the membranes were probed with the appropriate primary antibody in blocking buffer: N-cadherin (Invitrogen, 1:1,000), E-cadherin (Santa Cruz, 1:1,000), and β-actin (Abcam, 1:1,000) overnight at 4°C. After washing three times in TBS-T, membranes were incubated with the appropriate anti-mouse or anti-rabbit secondary antibody (1:2000) in blocking buffer for one h. The blots were visualized using Clarity Western ECL (Bio-Rad Laboratories).

### Immuno-localization of E- and N-cadherin in the UROtsa parent and As^+3^- and Cd^+2^-transformed UROtsa cells

For immuno-localization, the cells were grown in 24 well plates containing 12 mm glass coverslips at 37°C, 5% CO_2_. Cells at confluent density were then fixed and stained using previously described procedures [31, 32]. Confluent cultures were fixed in ice-cold 100% methanol for 3–5 min at -20°C. For E-cadherin, the cells were incubated with rabbit α-human E-cadherin primary antibody (Santa Cruz) at 2 μg/ml followed by incubation with 2 μg/ml of goat α-rabbit Alexa Fluor 594-tagged secondary antibody (Invitrogen). For N-cadherin, the cells were incubated with mouse α-human N-cadherin primary antibody at a concentration of 10 μg/ml followed by 2 μg/ml goat α-mouse secondary antibody Alexa Fluor 488 (Invitrogen). All antibody incubations were done at 37°C for 45 min each. Coverslips were mounted in ProLong Gold or Diamond anti-fade reagent with DAPI (Invitrogen) for nuclear counter staining. For the co-localization of N- and E-cadherin, cells were stained for E-cadherin followed by staining for N-cadherin. Cells were observed and images captured using a Leica TCS SPE, DM5500 laser scanning confocal microscope with LAS AF software (Leica Microsystems) and processed using Adobe Photoshop CS6. Images were obtained by capturing z-slices at the optimal depth of 0.347 mm. Controls consisted of coverslips treated with PBS instead of primary antibody. All controls stained appropriately and had virtually no specific staining when photographed under the same settings that were used for experimental cells.

### Immuno-histochemical localization of E- and N-cadherin in tumor transplants

The mouse subcutaneous heterotransplants were fixed in 10% neutral buffered formalin for 16–18 hours. All tissues were transferred to 70% ethanol and dehydrated in 100% ethanol. Dehydrated tissues were cleared in xylene, infiltrated, and embedded in paraffin. Tissue sections were cut at 3–5 μm for use in immuno-histochemical protocols. Prior to immuno-staining, sections were immersed in preheated citrate buffer pH 6.0 and heated in a steamer for 20 minutes. The sections were allowed to cool to room temperature and immersed into TBS-T (Dako, Carpinteria, CA) for 5 minutes. The immuno-staining was performed using the Dako ARK^TM^ (Animal Research Kit, K3954, Dako, Carpinteria, CA) following the manufacturer’s recommended protocol. The N-cadherin antibody (Life Technology) and the E-cadherin antibody (Santa Cruz Biotechnology) were used at a dilution of 1:100. Liquid diaminobenzidine was used for visualization. Slides were rinsed in distilled water, dehydrated in graded ethanol, cleared in xylene, and coverslipped. Normal human kidney and Dako Universal Negative were used as positive and negative controls.

### Flow cytometry

Cells were dissociated in 0.1% trypsin, 0.5 mM CaCl_2_ in PBS followed by the addition of 0.5 ml 10 mM EDTA. Non-specific staining was blocked using 1% bovine serum albumin (BSA) for 15 min on ice. *Phycoerythrin* (PE)-conjugated N-cadherin (mouse mAb clone 8C11, Biolegends cat # 350806) was added to cells at 1:10 dilution and incubated at 4°C in the dark for 30 min. Samples were washed two times in 0.1% BSA in PBS before flow cytometry analysis. Cells were counted with a BD LSRII using FACSDiva software. Results were gated to exclude doublets and identify the singlet population.

### Statistical analysis

All experiments were performed in triplicate and the results are expressed as the standard error of the mean. Statistical analyses were performed using GraphPad Prism® software using separate variance t-tests, ANOVA with Tukey post-hoc testing. Unless otherwise stated, the level of significance was 0.05.

## Results

### Expression of N-cadherin mRNA and protein in the parental UROtsa cell line and its As^+3^- and Cd^+2^-transformants

**Fig 1:**
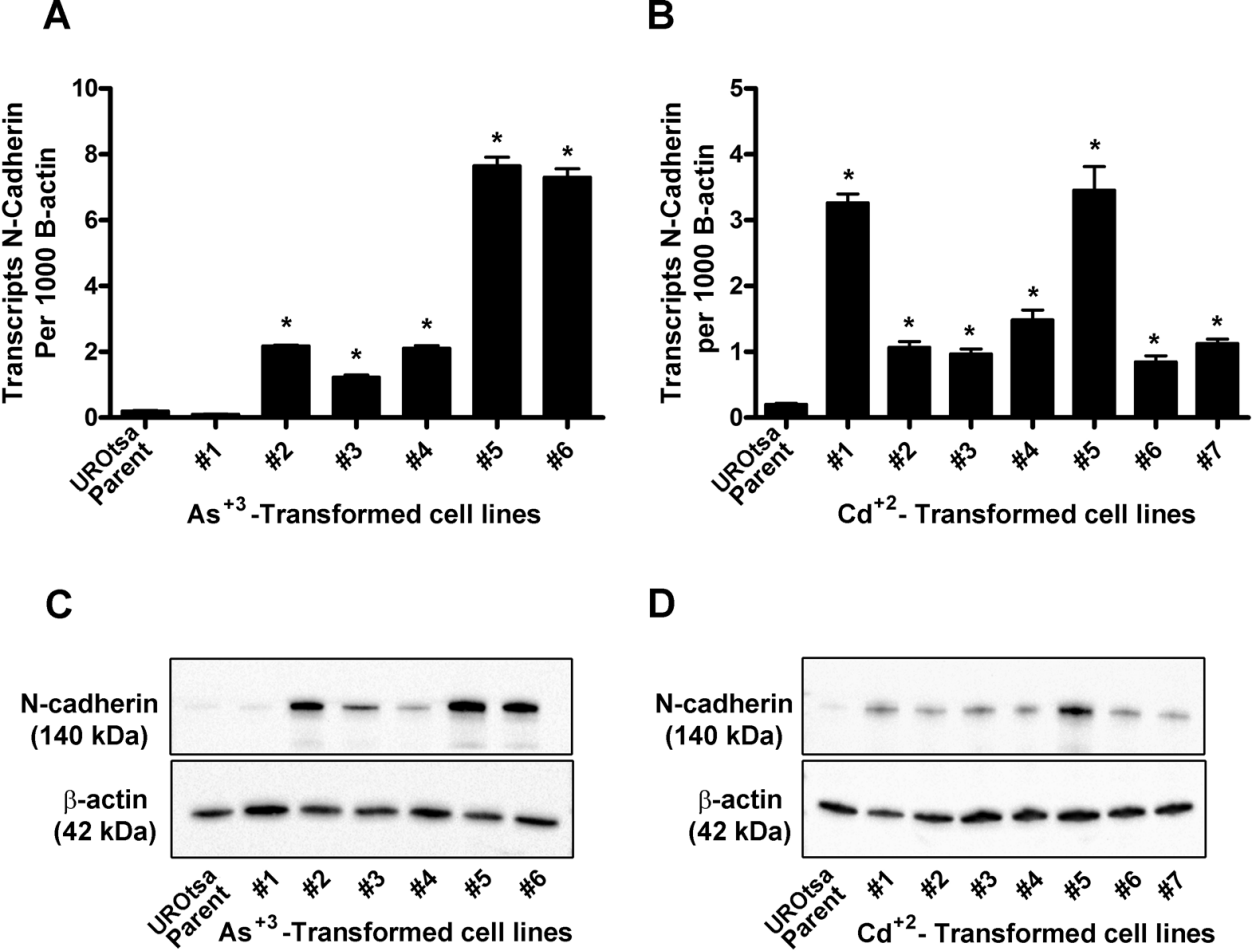
Expression of N-cadherin in the UROtsa parent and the As^+3^-and Cd^+2^-transformed cell lines. (A and B). Real time-RT-PCR analysis of N-cadherin expression. The data is expressed as the number of transcripts of N-cadherin per 1000 transcripts of β-actin and is plotted as the mean ± SEM of triplicate determinations. (C and D). Western analysis of N-cadherin protein. * denotes significant difference from UROtsa parent cells (p<0.05).

Total RNA was isolated from confluent cultures of parental UROtsa cells, the 6 independent isolates of As^+3^-transformed UROtsa cell lines, and the 7 independent isolates of the Cd^+2^-transformed UROtsa cell lines and analyzed for the expression of N-cadherin mRNA using real-time PCR. The results of this analysis showed a very low expression of N-cadherin mRNA in the parental UROtsa cells (Fig 1A). In contrast, N-cadherin mRNA expression was significantly elevated in 5 of the 6 As^+3^-transformed cell lines (Fig 1A) and in all 7 of the Cd^+2^- transformed cell lines (Fig 1B). The elevated expression of N-cadherin was variable in the transformed cell lines, varying between 5- and 40-fold in the As^+3^-transformed cell lines and between 4-and 18-fold in the Cd^+2^-transformed cell lines compared to the parental UROtsa cells. An analysis for the N-cadherin protein using Western blots demonstrated that the parental UROtsa cells had trace expression of the N-cadherin protein (Fig 1C). Identical with that found for the expression of N-cadherin mRNA, N-cadherin protein was expressed at elevated levels in protein lysates from 5 of the 6 As^+3^-transformed cell lines (Fig 1C) and in all 7 of the Cd^+2^- transformed cell lines (Fig 1D). The As^+3^-transformed cell line designated as isolate #1 had N-cadherin mRNA and protein expression levels similar to the parental UROtsa cells. For the other transformed isolates expressing N-cadherin, there was only a weak correlation between the levels of N-cadherin mRNA and protein expression for each individual cell line. Exposure of the parental UROtsa cells to either 1.0 μM of As^+3^ or Cd^+2^ for 30 days had no effect on the expression of N-cadherin mRNA or protein (data not shown). During this period of exposure, the cells did not form colonies in soft agar. This suggests that long term exposure does not induce the expression of N-cadherin and that the actual transformation process may be required to induce the expression of the gene.

### Expression of E-cadherin mRNA and protein in the parental UROtsa cell line and the As^+3^- and Cd^+2^-transformants

**Fig 2:**
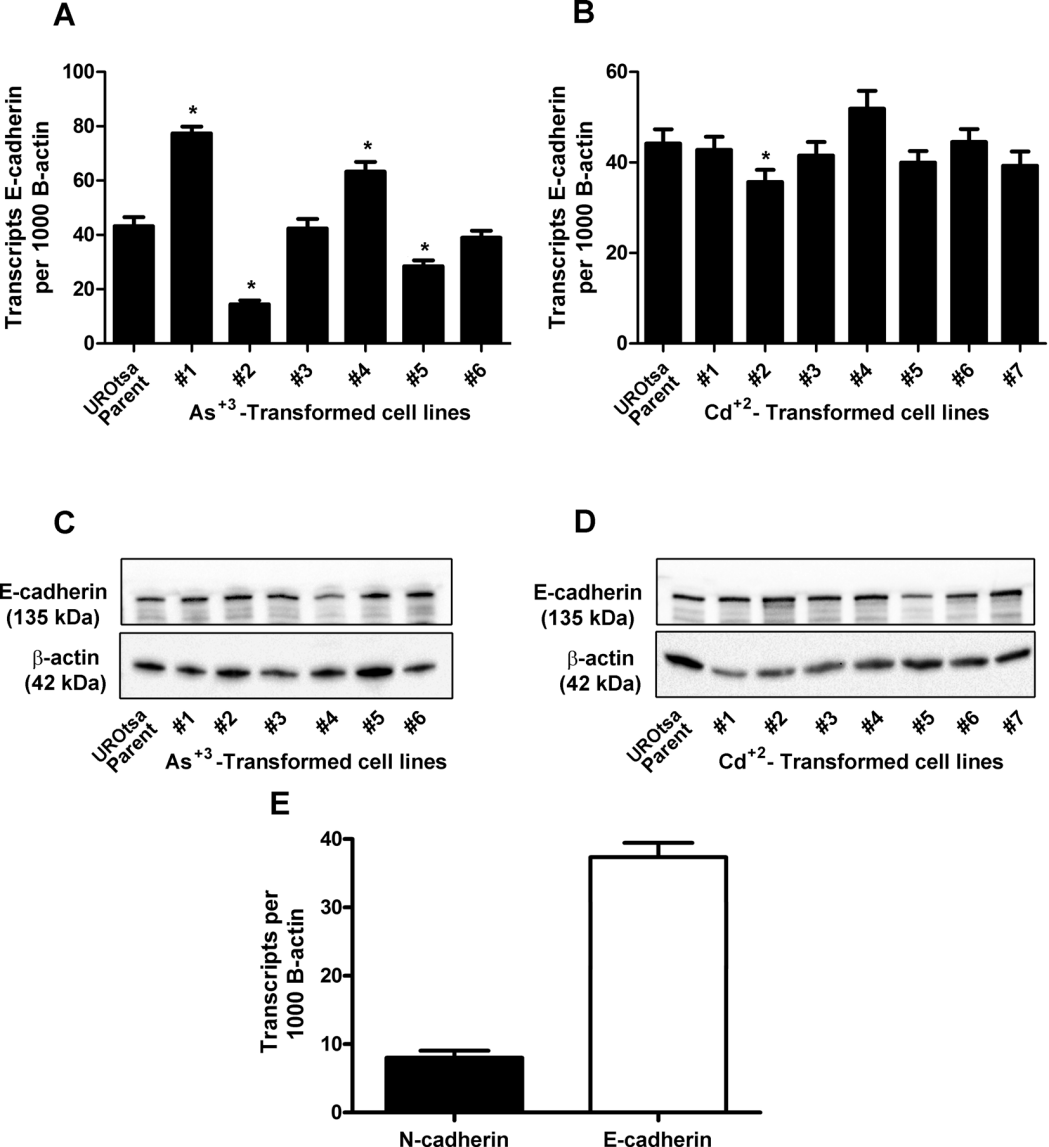
Expression of E-cadherin in the UROtsa parent and the As^+3^-and Cd^+2^-transformed cell lines. (A and B). Real time-RT-PCR analysis of E-cadherin expression. The data is expressed as the number of transcripts of E-cadherin per 1000 transcripts of β-actin and is plotted as the mean ± SEM of triplicate determinations. (C and D). Western analysis of E-cadherin protein. (E). Comparison of N and E-cadherin mRNA expression in As#6. * denotes significant difference from UROtsa parent cells (p<0.05).

Total RNA isolated from confluent cultures of parental UROtsa cells, the 6 independent isolates of As^+3^-transformed UROtsa cell lines, and the 7 independent isolates of the Cd^+2^-transformed UROtsa cell lines was also used to determine the expression of E-cadherin mRNA using real-time PCR. The results of this analysis showed that the parental UROtsa cell line as well as all the As^+3^- (Fig 2A) and Cd^+2^- transformed cell lines (Fig 2B) expressed E-cadherin mRNA. The level of E-cadherin mRNA was a common transcript in all the cell lines, being on the order of 200 transcripts per cell based on the common range of RNA amounts per cell being 10–30 pg/cell. The expression of E-cadherin mRNA was similar between the parental UROtsa cells and the 7 independent isolates of the Cd^+2^-transformed UROtsa cells. There was some heterogeneity in E-cadherin mRNA expression between the parental UROtsa cell line and the 6 As^+3^-transformed isolates, with 2 being equal, 2 reduced, and 2 increased when compared to the parental cell line. However, these differences in E-cadherin expression compared to parental cells, while statistically significant, were modest and within a one to two-fold range of variation. In general, the expression of the E-cadherin protein followed that of E-cadherin mRNA, with the levels being within a narrow range between the parental UROtsa cells and all the transformed isolates (Fig 2C and 2D). A quantitative comparison of E- and N-cadherin mRNA expression shows E-cadherin mRNA to be expressed at levels much higher than that of N-cadherin, as illustrated for the isolate (As#6) expressing the highest levels of N-cadherin mRNA (Fig 2E).

### Expression of N- and E-cadherin mRNA and protein in tumor transplants produced from As^+3^- and Cd^+2^-transformed UROtsa cells

**Fig 3:**
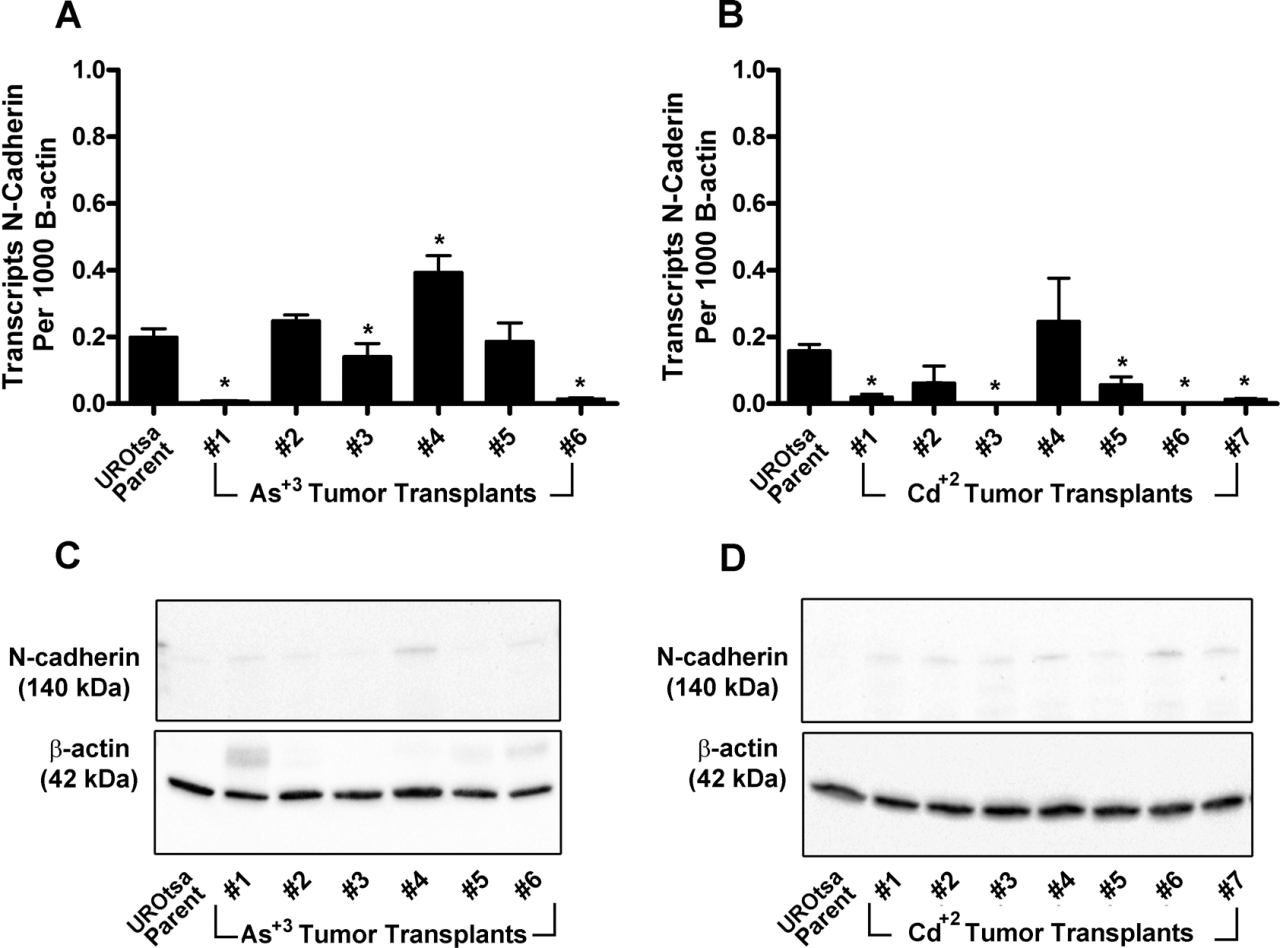
Expression of N-cadherin in the UROtsa parent cell line and the As^+3^ and Cd^+2^ tumor transplants. (A and B). Real time-RT-PCR analysis of N-cadherin expression. The data is expressed as the number of transcripts of N-cadherin per 1000 transcripts of β-actin and is plotted as the mean ± SEM of triplicate determinations. (C and D). Western analysis of N-cadherin protein. * denotes significant difference from UROtsa parent cells (p<0.05).

Subcutaneous tumors derived from all 6 As^+3^-transformed UROtsa cell lines and all 7 Cd^+2^-transformed cell lines were analyzed for the expression of N- and E-cadherin mRNA and protein. The N- and E-cadherin protein was determined by both Western blotting and immuno-histochemistry. For N-cadherin, the results of this analysis showed that N-cadherin mRNA was expressed at very low levels in total RNA isolated from any of the tumor transplants produced by the As^+3^-and Cd^+2^-transformed cell lines (Fig 3A and 3B, respectively). The levels of N-cadherin expression were similar to that found in the parental UROtsa cell line and would be on the order of less than 1 transcript of N-cadherin per cell. Western blotting for expression of the N-cadherin protein showed that there was only a background level of N-cadherin expression in lysates prepared from tumors from all 6 As^+3^-and all 7 Cd^+2^-transformed cell lines (Fig 3C and 3D, respectively).

**Fig 4:**
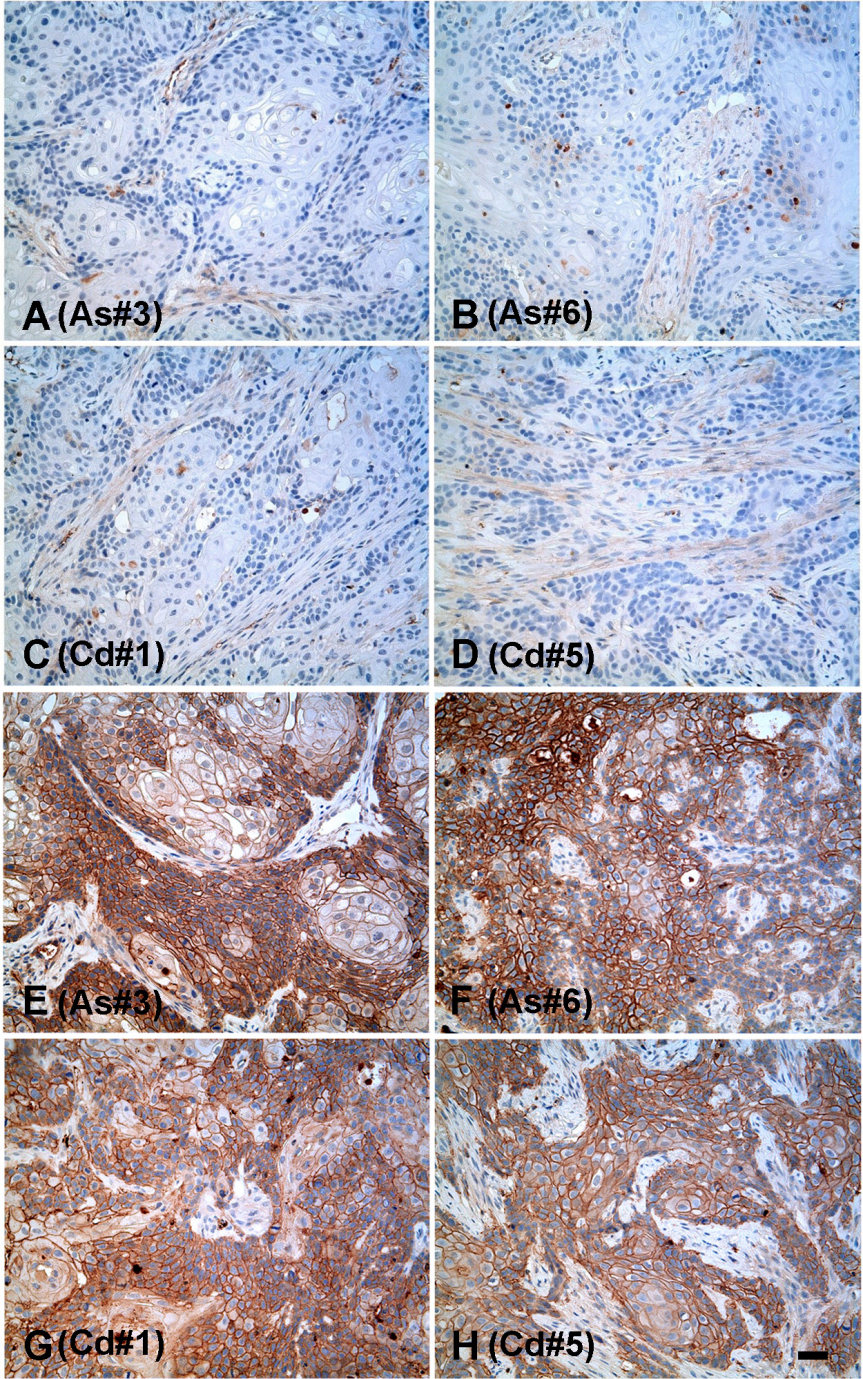
Immunohistochemical analysis of N and E-cadherin protein in tumor transplants. (A-D). Expression of N-cadherin protein in tumor transplants generated from As#3, As#6, Cd#1 and Cd#5 cell lines respectively. (E-H) Expression of E-cadherin protein in tumor transplants generated from As#3, As#6, Cd#1 and Cd#5 cell lines respectively. The brown color indicates the presence of the protein whereas the blue color indicates the nuclei that were stained with the counterstain hematoxylin. All images are at a magnification of X200. Scale bar = 100 μm and is shown for panels A-H.

Immuno-histochemistry was also employed to determine the localization and expression of N-cadherin protein in the tumors produced from the As^+3^-and Cd^+2^-transformed cell lines. It was shown that none of the tumors produced from the 6 As^+3^-and 7 Cd^+2^-transformed cell lines stained for the presence of the N-cadherin protein. While tumors produced from all 6 As^+3^-and 7 Cd^+2^-transformed cell lines failed to stain for the N-cadherin protein, only tumors produced from the As#3 and As#6, Cd#1 and Cd#5 cell lines are shown for illustration (Fig 4A–4D, respectively). These 4 examples were chosen since they covered the spectrum of N-cadherin expression, from lowest to highest, in the transformed cell lines. Higher magnification of the figure is provided in the supporting information section (S1 Fig).

**Fig 5:**
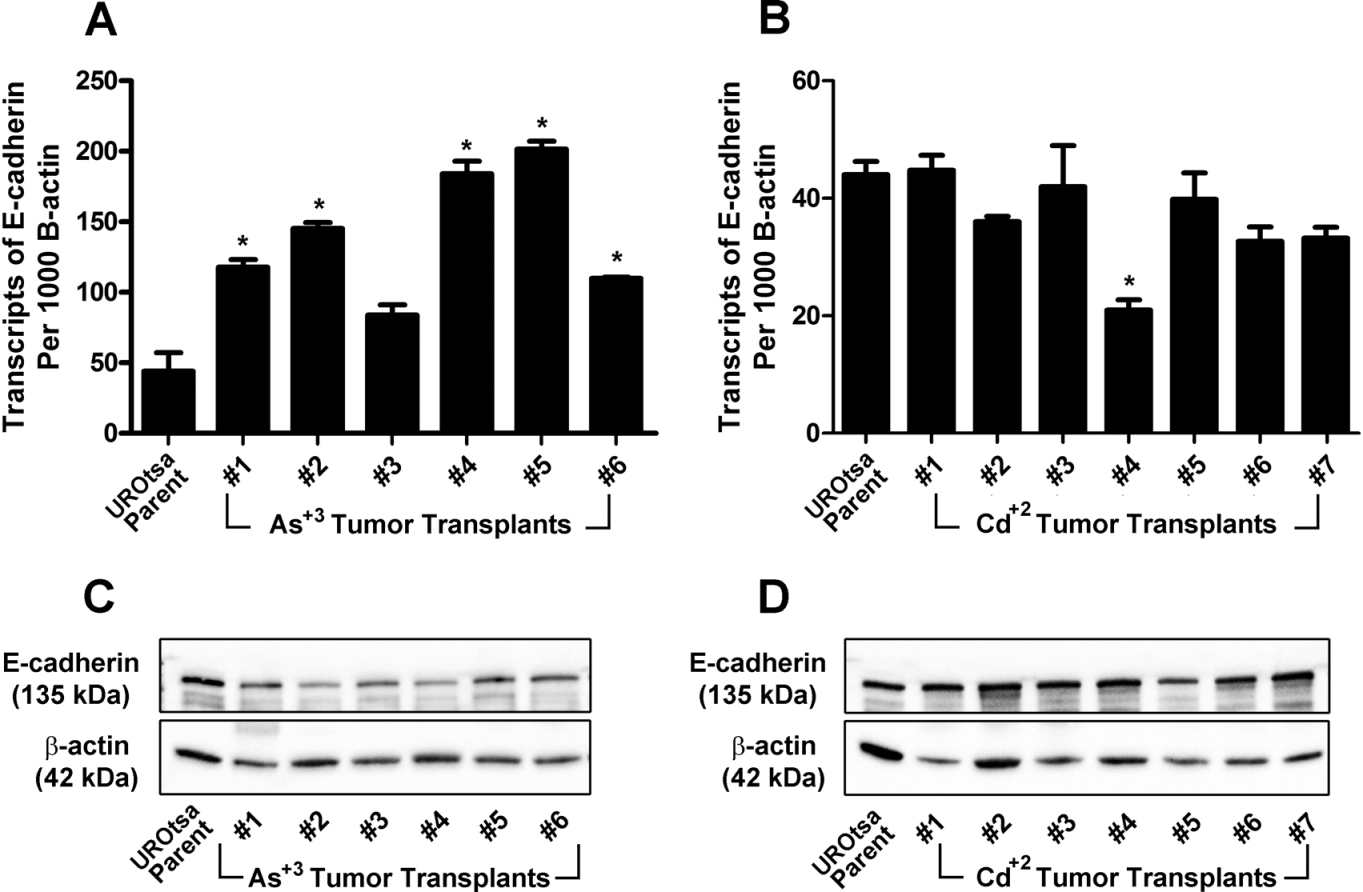
Expression of E-cadherin in the UROtsa parent cell line and the As^+3^ and Cd^+2^ tumor transplants. (A and B). Real time-RT-PCR analysis of E-cadherin expression. The data is expressed as the number of transcripts of E-cadherin per 1000 transcripts of β-actin and is plotted as the mean ± SEM of triplicate determinations. (C and D). Western analysis of E-cadherin protein. * denotes significant difference from UROtsa parent cells (p<0.05).

For E-cadherin, the results showed that all the tumor transplants produced by the As^+3^- (Fig 5A) and Cd^+2^-(Fig 5B) transformed cells expressed E-cadherin mRNA at levels equal to or greater than that found in the parental UROtsa cell line. The level of E-cadherin mRNA was similar in all the tumors produced by the Cd^+2^-transformed cell lines, however the levels were higher and more variable in tumors produced from the 6 As^+3^-transformed cell lines. Western blotting for the expression of the E-cadherin protein showed that the tumors from all the As^+3^- (Fig 5C) and Cd^+2^ (Fig 5D) -transformed cell lines expressed the E-cadherin protein. Immuno-histochemistry was used to determine the localization and expression of E-cadherin protein in the tumors produced from the As^+3^-and Cd^+2^-transformed cells. It was shown that all of the tumors produced from the 6 As^+3^-and 7 Cd^+2^-transformed cell lines stained intensely for the presence of the E-cadherin protein and the tumors produced from the As#3 and As#6, Cd#1 and Cd#5 isolates are shown for illustration (Fig 4E–4H, respectively).

### Immuno-localization of N-and E-cadherin in parental UROtsa cells and the As^+3^- and Cd^+2^-transformed UROtsa cells

**Fig 6:**
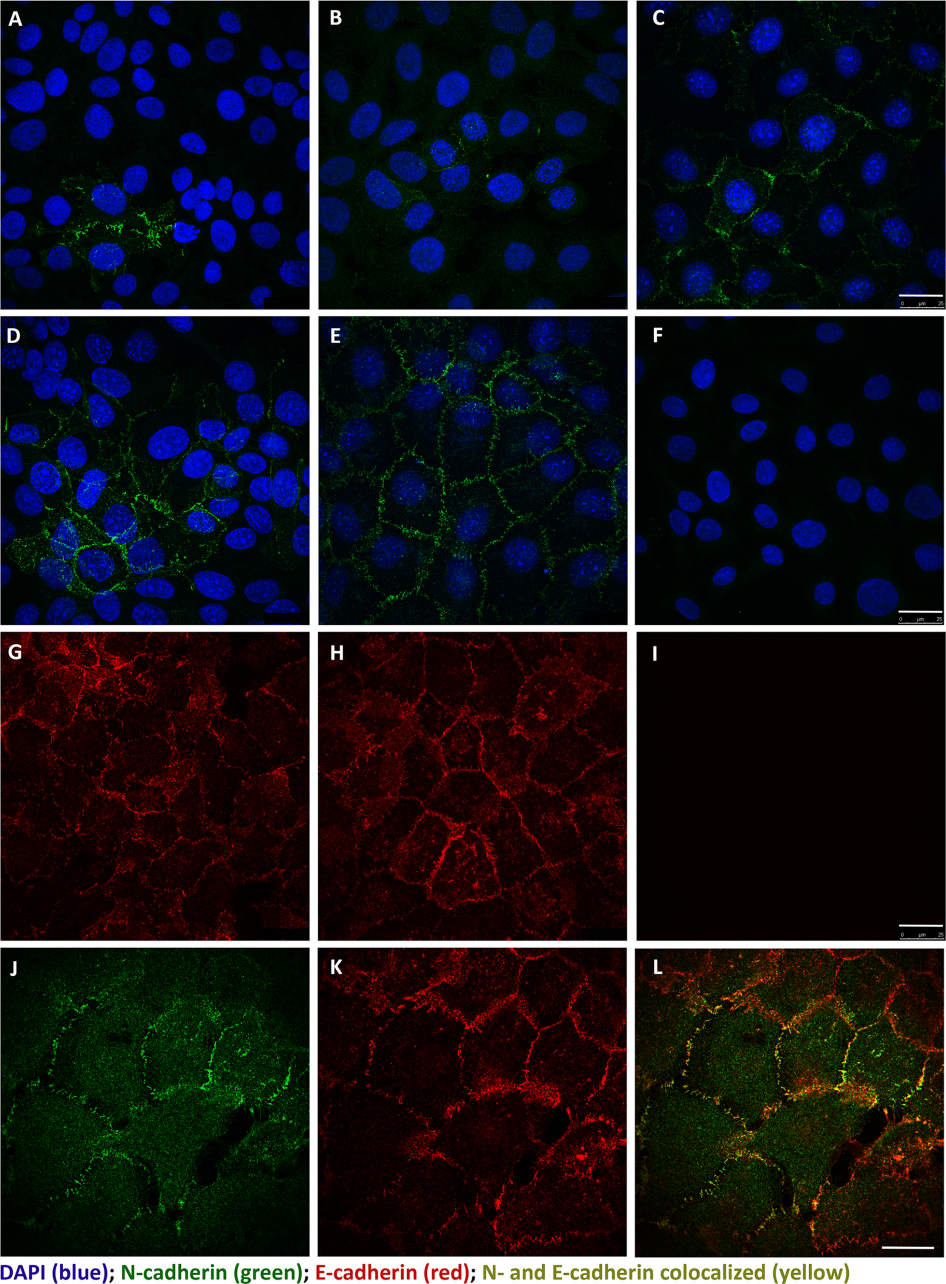
Immunofluorescent localization of N- and E-cadherin in UROtsa cells by scanning confocal microscopy. N-cadherin (green) was found to be localized to the plasma membrane, when present, in the parent and transformed UROtsa cell lines: (A) UROtsa parent; (B) Cd#1; (C) Cd#5; (D) As#3; (E) As#6 with DAPI shown as a counterstain. A representative negative control that lacks primary antibody is shown in (F) with the UROtsa Parent cell line. E-cadherin (red) was also found to be localized to the plasma membrane and present in nearly all of the cells within the population. Representative images are shown for UROtsa Parent (G) and As#6 (H) cell lines. Likewise, a representative negative control that lacks primary antibody is shown in (I) for the UROtsa parent cells. To determine if N- and E-cadherin co-localized within the same cells, a single 0.347 μm z-plane image is shown for As#6 (J-L). N-cadherin (green) is shown in (J), E-cadherin (red) is shown in (K), and the merged images showing co-location of N- and E-cadherin (yellow) is shown in (L). Both scale bars = 25 μm and are shown in (I) for panels A-I and in (L) for panels J-L.

Confocal microscopy was used to determine the intracellular localization and prevalence of N- and E-cadherin in the parental UROtsa cells and their As^+3^-and Cd^+2^-transformed counterparts. The localization of N-cadherin in the parental UROtsa cells showed that over 85% of the cells exhibited no staining for the N-cadherin protein (Fig 6A). In the few profiles where N-cadherin staining was present, there were two profiles of cells, one population that was weakly stained and another population with strong staining for N-cadherin. When present, N-cadherin was localized to the plasma membrane. The localization of N-cadherin in the 7 Cd^+2^- transformed UROtsa cell lines showed a wide variation in the percentage of the population reactive for N-cadherin ranging from less than 5% of the cells positive for N-cadherin at the plasma membrane (Cd#1, Cd#7) to moderately positive for 20–40% of the population (Cd #2, #3, #4, and #6) to greater than 60% moderately positive (Cd#5) (Fig 6B and 6C illustrated for the Cd#1 and Cd#5 cell lines, respectively). Similarly, the localization of N-cadherin in the 6 As^+3^- transformed cell lines showed the same range of low to high overall N-cadherin staining at the plasma membrane. One of the isolates had very low staining (As#1), three of the 6 isolates had moderate levels of staining with some cells staining intensely (As#2, #3, #4) and two isolates had high levels, greater than 85% of the population, with many intensely staining cells (As#5, #6) (Fig 6D and 6E illustrated for the As#3 and As#6 cell lines with a representative control of no primary antibody shown in F). Higher magnification of the figure is provided in the supporting information section (S2 Fig).

Flow cytometry was used to confirm these visual observations for the UROtsa parent and 4 of the cell lines. For UROtsa parent cells, 58% of the cells had moderate expression of N-cadherin, whereas less than 2% of the cells had high expression of N-cadherin. For the As^+3^-transformed cell lines, approximately 98% of the cells of the As#6 cell line and 40% of the cells of the As#3 cell line showed strong to moderate expression of N-cadherin respectively. The Cd#1 and Cd#5 cell lines showed moderate expression in about 40 and 60% of the cells respectively (Table 1).

**Table 1:** Flow analysis of N-cadherin expressing UROtsa cells.

Isolate	Cells (Number)	Low N-cadherin (Number)	Low N-cadherin (Percent)	Moderate N-cadherin (Number)	Moderate N-cadherin (Percent)	High N-cadherin (Number)	High N-cadherin (Percent)
UROtsa	4437	1677	37.8	2583	58.2	58	1.31
As#3	7317	3840	52.5	2982	40.8	117	1.6
As#6	7536	2	0.027	9	0.12	7426	98.5
Cd#1	7140	3325	46.6	3207	44.9	101	1.41
Cd#5	6868	1866	27.2	4354	63.4	60	0.87

Immuno-fluorescence was also performed for the localization of E-cadherin in the parental UROtsa cells and in two each of the As^+3^-and Cd^+2^-transformed counterparts (As#3, As#6, Cd#1, Cd#5). In all 5 cell lines, E-cadherin staining was uniformly intense, localized to the plasma membrane, and expressed by all the cells in the monolayer’s examined for each cell line (Fig 6G and 6H- illustrated for the parental UROtsa cells and the As#6 cell line with a representative control of no primary antibody shown in I). The co-localization of E- and N-cadherin was also determined on the As#6 cell line since it had the highest expression of N-cadherin. The results showed that E- and N-cadherin both localized to the plasma membrane and that both exhibited strong staining in cells where they were co-localized (Fig 6J, 6K and 6L). Flow cytometry showed that all of the 5 cell lines expressed high levels of E-cadherin protein (data not shown).

### Expression of N-cadherin in cancer initiating cell (CIC) spheroids and resultant tumor transplants

**Fig 7:**
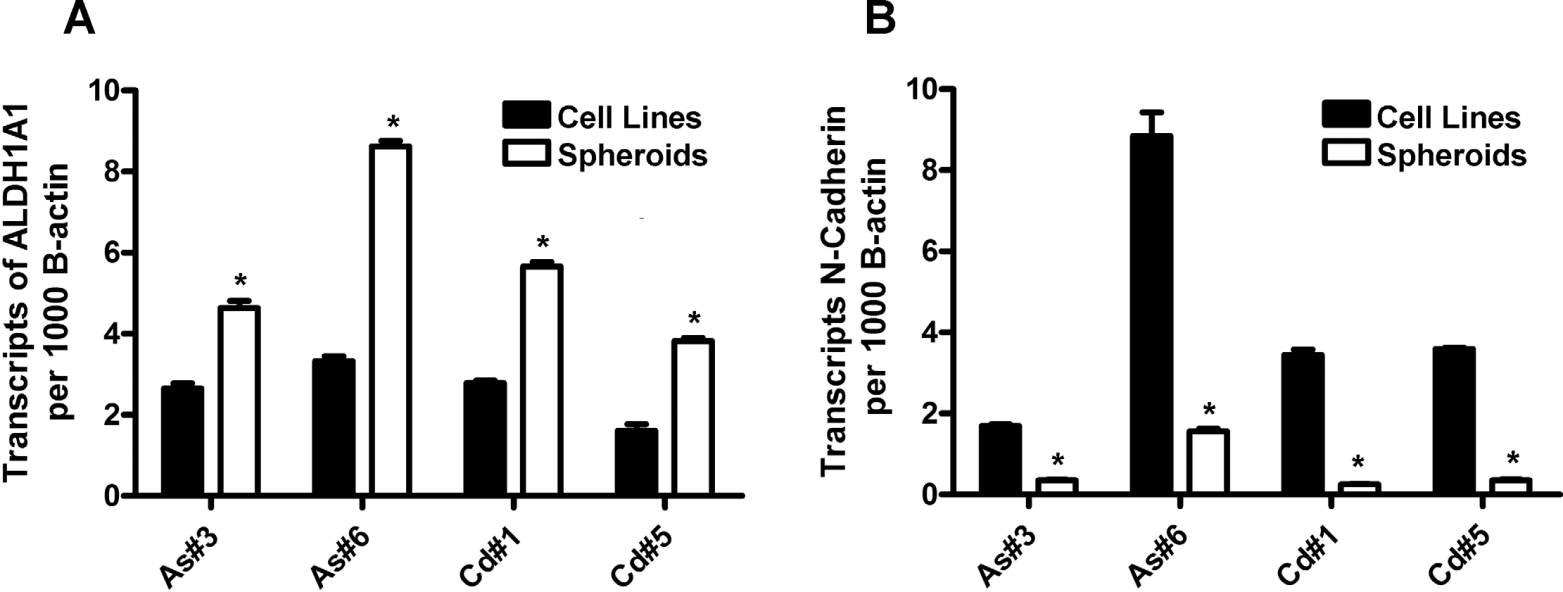
Expression of ALDH1A1and N-cadherin in cancer initiating cell spheroids. (A and B). Real time-RT-PCR analysis of ALDH1A1 and N-cadherin expression. The data is expressed as the number of transcripts per 1000 transcripts of β-actin and is plotted as the mean ± SEM of triplicate determinations. (C and D). * denotes significant difference from corresponding parental cell line (p<0.05).

Spheroids were prepared from As#3, As#6, Cd#1 and Cd#5 cell lines and total RNA was assessed for the expression of aldehyde dehydrogenase 1A (ALDH1A), a stem cell marker, and N-cadherin mRNA. It was demonstrated that spheroids from all 4 cell lines had an elevated expression of ALDH1A when compared to their parent cell lines (Fig 7A). In contrast, it was demonstrated that the spheroids from all 4 cell lines had a reduced expression of N-cadherin mRNA when compared to their parent cell lines (Fig 7B).

**Fig 8:**
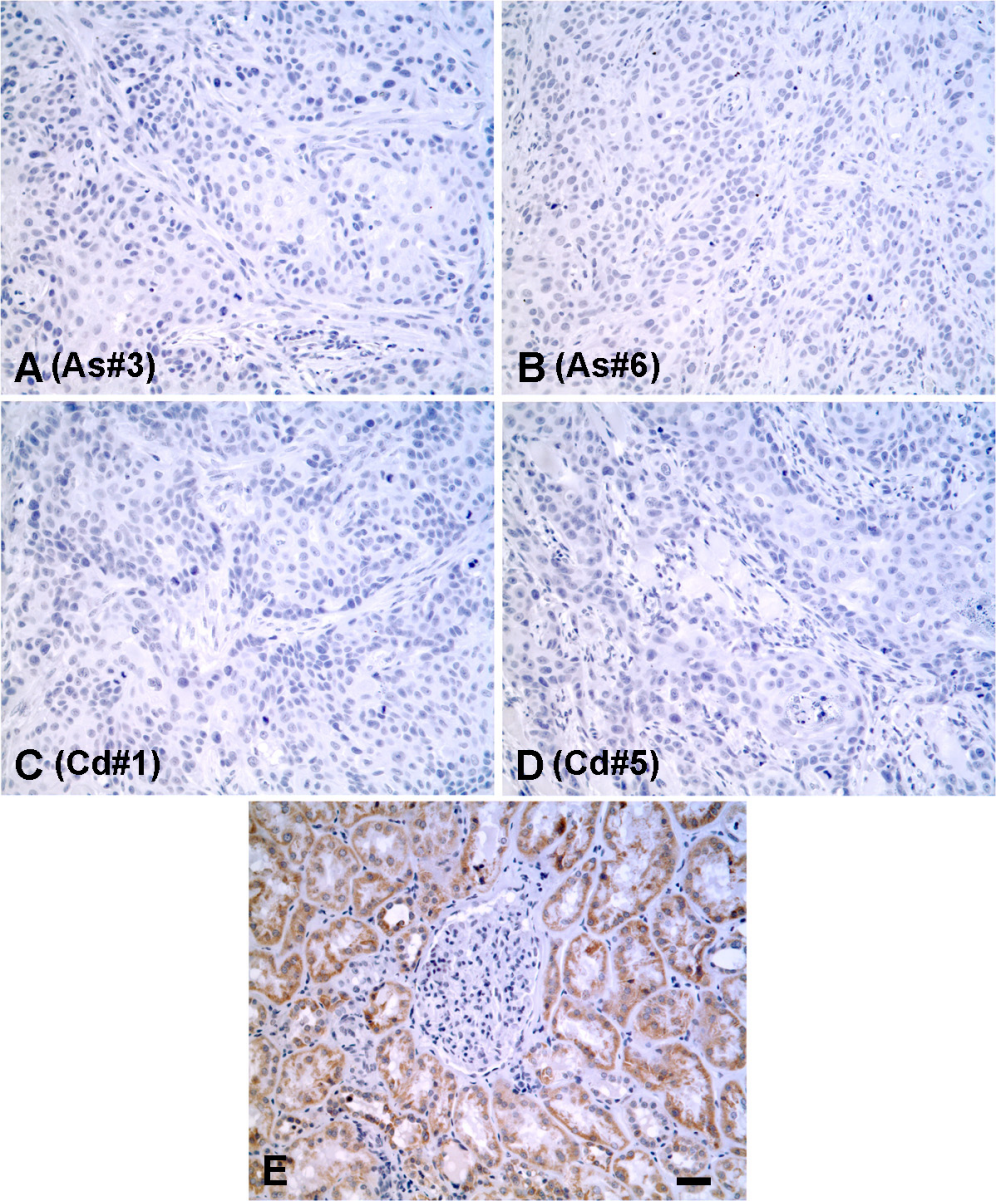
Immunohistochemical analysis of N-cadherin protein in tumor transplants generated from cancer initiating cell spheroids. (A-D). Expression of N-cadherin protein in tumor transplants generated from As#3, As#6, Cd#1 and Cd#5 cancer initiating cell spheroids respectively. (E). Human kidney stained for N-cadherin as a positive control. The brown color indicates the presence of the protein whereas the blue color indicates the nuclei that were stained with the counterstain hematoxylin. All images are at a magnification of X200. Scale bar = 100 μm and is shown for panels A-E.

Tumor transplants were generated from the above 4 spheroids and assessed for their expression of N-cadherin using immuno-histochemistry. It was demonstrated that none of the tumor transplants generated from the spheroids stained for the N-cadherin protein (Fig 8A–8D). Higher magnification of the figure is provided in the supporting information section (S3 Fig). The histology of the transplants generated from the spheroids was identical to that generated from the parental cell lines (Fig 8A–8D versus Fig 4A–4D).

### Expression of N-cadherin and E-cadherin in the J82, RT4 and TCCSUP human urothelial cancer cell lines

**Fig 9:**
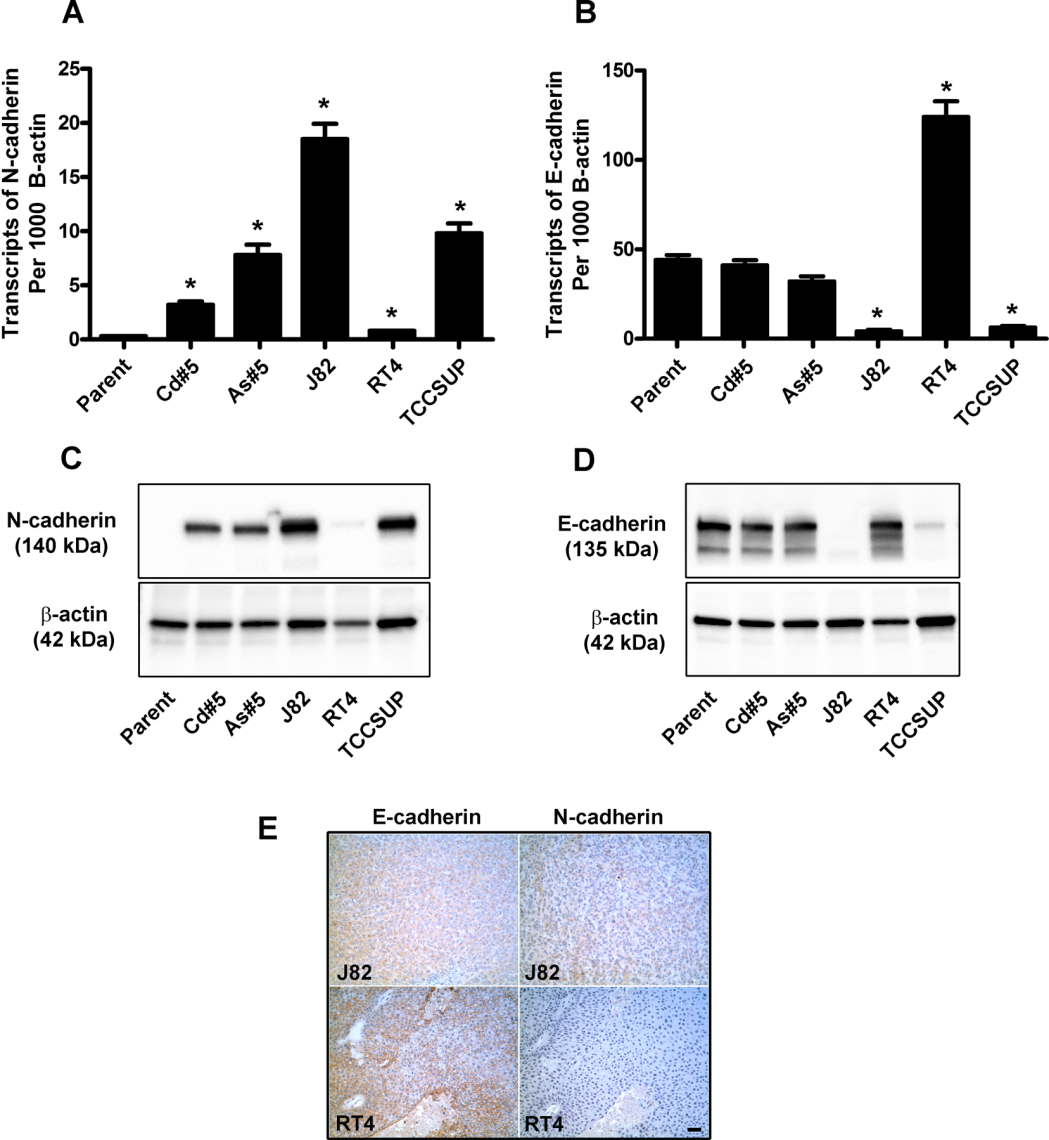
Expression of N and E-cadherin in J82, RT4 and TCCSUP urothelial cancer cell lines and tumor transplants. (A and B). Real time-RT-PCR analysis of N and E- cadherin expression. The data is expressed as the number of transcripts of N and E-cadherin per 1000 transcripts of β-actin and is plotted as the mean ± SEM of triplicate determinations. (C and D). Western analysis of N and E-cadherin protein. * denotes significant difference from UROtsa parent cells (p<0.05). The expression level of N and E-cadherin in As#5 and Cd#5 are included for comparison. (E). Immuno-histochemical analysis of N and E-cadherin protein in tumor transplants. The brown color indicates the presence of the protein whereas the blue color indicates the nuclei that were stained with the counterstain hematoxylin. All images are at a magnification of X200. Scale bar = 100 μm.

The expression of N-cadherin and E-cadherin mRNA and protein was determined by real-time PCR and western blotting in 3 commonly utilized urothelial cancer cell lines. It was demonstrated the N-cadherin mRNA (Fig 9A) and protein (Fig 9C) were elevated in the J82 and TCCSUP cell lines when compared to the RT4 cell line. In contrast, E-cadherin mRNA (Fig 9B) and protein (Fig 9D) were elevated in the RT4 cell line when compared J82 and TCCSUP cell lines. The elevated expression of E-cadherin and low expression of N-cadherin in the RT4 cell line corresponds to its epithelial morphology while the converse is true for the J82 and TCCSUP cell lines which have a mesenchymal morphology (ATCC data sheet). Immuno-histochemistry was used to determine the localization and expression of E and N-cadherin in the tumors produced by the subcutaneous injection of J82 and RT4 cell lines (TCCSUP did not form a tumor). The results (Fig 9E) showed that E-cadherin was expressed in the tumors produced by both the J82 and the RT4 cell lines although the expression was more intense in the tumors produced by the RT4 cell lines. The expression of N-cadherin was weak in the tumor produced by the J82 cell line, whereas the tumors produced by the RT4 cell line did not express N-cadherin. Higher magnification of the figure is provided in the supporting information section (S4 Fig).

## Discussion

The initial goal of this study was to show that N-cadherin expression was increased in UROtsa cells malignantly transformed by As^+3^ and Cd^+2^, providing evidence that the transformed cells were undergoing EMT. Evidence that N-cadherin would be elevated in the As^+3^-and Cd^+2^-transformed cell lines came from an earlier microarray analysis of 6 independent isolates of the As^+3^-transformed cell lines and 5 independent isolates of the Cd^+2^-transformed cell lines [33]. The multiple independent isolates of the As^+3^-and Cd^+2^-transformed cells were isolated to determine the degree of heterogeneity in gene expression that might occur among isolates malignantly transformed using identical conditions of exposure to the toxicant. The analysis of the expression patterns of these cell lines using the Affymetrix 133 plus 2.0 chip showed that N-cadherin was the most induced gene in the As^+3^-transformed cell lines (56.9 fold) and the 5^th^ most induced gene in the Cd^+2^-transformed cell lines (34.9 fold). Based on this earlier data it was expected that each individual cell line would demonstrate an increase in the expression of N-cadherin mRNA and protein. As expected, an increase in overall N-cadherin mRNA and protein was confirmed by real-time PCR and western blotting for each cell line. However, immuno-localization studies on N-cadherin showed a marked heterogeneity within each cell line regarding the % of cells that were leading to the increased N-cadherin expression noted by real-time PCR and western blotting. This was especially noteworthy for two of the seven Cd^+2^-transformed cell lines and one of the six As^+3^-transformed cell lines where less than approximately 5% of the cells showed reactivity for N-cadherin while four of the Cd^+2^-and three of the As^+3^-transformed cell lines demonstrated moderate to strong N-cadherin reactivity between 20 and 50% of the cells. However, the transformed cell lines with the highest expression of N-cadherin via Western analyses, As#5, As#6, and Cd#5 had not only cells stained moderately to strongly for N-cadherin at the plasma membrane but also had between 60–90% of the population positive for N-cadherin. Thus, these results suggest that the expression level of N-cadherin by real-time PCR and western analysis for the overall culture is not necessarily indicative of the expression levels of the individual cells in the culture. This has several implications on the interpretation of results from individual cell cultures displaying this degree of heterogeneity. One is that the level of N-cadherin expression in the cells that do express N-cadherin is often much higher than would be expected from real-time PCR and western blotting of the entire culture. This is especially true for the cell lines where less than 15% of the cells are expressing N-cadherin. This is important since it does support the concept that this minority population of cells is undergoing the process of EMT and possibly cadherin switching. The second implication is more practical and it is the limitation that such heterogeneity within an individual culture places on the use of such a culture system for mechanistic studies. In the present case, mechanistic studies on the regulation of N-cadherin could be very misleading since, in many of the cell lines, the majority of the cells do not express N-cadherin even though N-cadherin mRNA and protein can be determined for each line. This is especially noteworthy in the present study since a microarray analysis of the cell lines indicated that N-cadherin was one of the most highly induced genes and the cell cultures studies using real-time PCR and western blotting were supportive of the finding of induced expression.

The finding of the heterogeneity in gene expression among the individual independent isolates of the As^+3^-and Cd^+2^-transformed cell lines is expected as a hallmark of malignant transformation. However, the finding of variation of N-cadherin expression within the individual cells comprising each cell line was not expected. The finding that N-cadherin expression was variable among the cells comprising each cell line appears to be unique and not a reflection of a cell culture system or a series of cell lines that are composed of a variety of cells with diverse expression profiles. The expression of E-cadherin was determined in the present study and expression was uniform within all the cell lines and among the individual cell lines. There were only very rare profiles of cells from any of the cell lines that did not express E-cadherin. Co-localization was performed for N-cadherin and E-cadherin in the As#6 cell line where the majority of cells express N-cadherin and all the cells that expressed N-cadherin also expressed E-cadherin. This finding also suggested that while N-cadherin expression was elevated, there was no evidence of “cadherin switching” since there was no noticeable reduction in E-cadherin expression. To assure that the reagents and methods utilized accurately detected E- and N-cadherin, their expression was also determined in 3 commonly used human urothelial cancer cell lines. The results showed that those cell lines know to have mesenchymal character showed elevated expression of N-cadherin and only background expression of E-cadherin and vice versa. Other studies on the UROtsa cell lines have shown a uniform expression of proteins within the cells of each line; SPARC in the parental UROtsa cells [35], and enolase 1, enolase 2, keratin 17, 19 and Zip8 in all the cell lines [32, 34–38]. One can speculate on why N-cadherin might be present in only a portion of the cells comprising each transformed UROtsa cell line. One explanation is that N-cadherin expression is increasing over time in each individual cell culture as the culture progresses to a more mesenchymal state. This would be in line with the widely accepted view that EMT leads to a cancer cell that has increased proliferation [39]. EMT might also be favored to occur due to the serum-containing growth media used in cell culture that would be expected to favor mesenchymal cell growth. Thus, an explanation to explain the finding that each As^+3^-and Cd^+2^-transformed cell line is composed of both cells positive and negative for the expression of N-cadherin is that each culture is a snap shot in time as each cell line progresses to one containing a more homogeneous population of cells expressing N-cadherin. This might also suggest that cells transformed by As^+3^ have a greater tendency for progression since N-cadherin was more pronounced in the As^+3^-transformed cell lines.

The tumor transplants produced from the 6 isolates of As^+3^-transformed cells and the 7 isolates of Cd^+2^-transformed cells showed no staining for the N-cadherin protein when examined by immune-histochemistry. Multiple slides from each tumor were examined to rule out the possibility of areas of focal staining for N-cadherin. Sections of human kidney were included as a control and showed the staining pattern known for N-cadherin. An examination of N-cadherin protein expression in tumor lysates by Western blotting also showed only background staining for N-cadherin. The finding that the tumor cells comprising the tumor transplants had no expression of N-cadherin was somewhat surprising since all the cell lines showed some expression of N-cadherin and the As#6 cell line showed the majority of cells as staining positive for N-cadherin on both immune-fluorescence examination and by flow cytometry. In contrast, all the tumors from all the cell lines stained intensely and uniformly for E-cadherin. One explanation for this finding would be that the cells expressing N-cadherin are not able to establish (seed) a tumor in the immune compromised mice following subcutaneous injection. This explanation would be in agreement with the classical view of EMT. In this view, it is widely accepted that the process of EMT is associated with a cell’s ability to escape the basement membrane and disseminate into the lymph nodes, blood stream or local body cavity [40–42]. On the other hand, MET is viewed as necessary for a cell that has undergone EMT and escaped into the extracellular environment to establish or “seed” a new metastatic site for growth [40–42]. In this view, the lack of N-cadherin expression in tumor transplants of the As^+3^-and Cd^+2^-transformed cells would be consistent with E- and not N-cadherin expressing cells “seeding” a site for tumor growth. For this explanation to be viable, the As^+3^- and Cd^+2^- transformed cell lines should possess a subpopulation of cells capable of initiating tumor formation, or what is commonly called a population of cancer-initiating cells (CICs) [43–45]. This was tested in the present study by isolating spheroids from 2 of the As^+3^- and Cd^+2^- transformed cell lines as a putative subpopulation of cells (CICs) that would be able to establish tumors in immune compromised mice. It was demonstrated that these CICs had only background levels of N-cadherin mRNA, elevated levels of ALDH1 mRNA and were able to establish tumors in immune compromised mice. The tumors produced by the injection of these CICs subcutaneously showed no staining for the N-cadherin protein by immuno-histochemical examination and the tumors displayed histologies indistinguishable from those produced by injection of the original cell lines. This is especially noteworthy for the CICs isolated from the As#6 cell line since it was composed almost entirely of cells that expressed N-cadherin. These findings provide strong supporting evidence that the cells that expressed N-cadherin in the As^+3^- and Cd^+2^-transformed cell lines were unable to “seed” tumors in the immune compromised mice. An alternate explanation would be that the CICs simply lose N-cadherin expression when they are unable to attach to the surface of the cell culture flask. However, this explanation is less likely since it would be surprising that these cells would not regain expression of N-cadherin once the cells comprised the tumor mass. The As^+3^ and Cd^+2^ tumor transplants did not grow back when put into cell culture, therefore it is not known if they would regain the ability to express N-cadherin when grown in a serum containing environment. The expression of E and N-cadherin in the tumors produced by the commercially available cell lines was also determined and it was found that the expression of E-cadherin was moderate to high, whereas the expression of N-cadherin was weak in the tumors produced by the J82 cell line and was absent in the tumors produced by the RT4 cell line. Thus this data recapitulates the results of the tumors produced by the As^+3^-and the Cd^+2^-transformed cell lines.

The results also serve as a caution for laboratories using cell culture models of human cancer, especially when they are used to produce tumor transplants. The results of the present study suggest that the population of cultured cells producing a tumor transplant may be very rare and distinct from the expression pattern of the overall culture.

## Conclusion

Urothelial cells transformed by the environmental toxicants arsenite and cadmium express N-cadherin *in-vitro*, however tumors formed by the subcutaneous injection of these transformed cells do not express N-cadherin. This finding is in agreement with the classical view of EMT where it is thought that the expression of N-cadherin is associated with the dissemination of the tumor, whereas the expression of E-cadherin is associated with tumor seeding. In addition, the study also shows that a small proportion of cells present in the culture are undergoing the process of EMT and possibly cadherin switching.

## Supporting Information

S1 Fig High Magnification of immunohistochemical analysis of N and E-cadherin protein in tumor transplants. (A-D). Expression of N-cadherin protein in tumor transplants generated from As#3, As#6, Cd#1 and Cd#5 cell lines respectively. (E-H) Expression of E-cadherin protein in tumor transplants generated from As#3, As#6, Cd#1 and Cd#5 cell lines respectively. The brown color indicates the presence of the protein whereas the blue color indicates the nuclei that were stained with the counterstain hematoxylin. All images are at a magnification of X400. Scale bar = 50 μm and is shown for panels A-H.
(TIF)

S2 Fig High magnification immunofluorescent localization of N- and E-cadherin in UROtsa cells by scanning confocal microscopy. N-cadherin (green) was found to be localized to the plasma membrane, when present, in the parent and transformed UROtsa cell line: (A, A’) UROtsa parent and (B, B’) As#6. Co-localization (yellow) of N-cadherin (green) and E-cadherin (red) is also shown (C, C’). High magnifications of the selected pictures are shown in the panels to the right (A’, B’, C’). The regions magnified are indicated by the white boxes on the lower magnification images. All scale bars = 10 μm.
(TIF)

S3 Fig High magnification of immunohistochemical analysis of N-cadherin protein in tumor transplants generated from cancer initiating cell spheroids. (A-D). Expression of N-cadherin protein in tumor transplants generated from As#3, As#6, Cd#1 and Cd#5 cancer initiating cell spheroids respectively. (E). Human kidney stained for N-cadherin as a positive control. The brown color indicates the presence of the protein whereas the blue color indicates the nuclei that were stained with the counterstain hematoxylin. All images are at a magnification of X400. Scale bar = 50 μm and is shown for panels A-E.
(TIF)

S4 Fig High magnification of immuno-histochemical analysis of N and E-cadherin protein in tumor transplants. The brown color indicates the presence of the protein whereas the blue color indicates the nuclei that were stained with the counterstain hematoxylin. All images are at a magnification of X400. Scale bar = 50 μm.
(TIF)
