# Supplementary figures and images for: Loss of N-Cadherin Expression in Tumor Transplants Produced From As+3- and Cd+2-Transformed Human Urothelial (UROtsa) Cell Lines
Source: PLoS One. 2016 May 25;11(5):e0156310. doi: 10.1371/journal.pone.0156310 (PMC4880289; doi:10.1371/journal.pone.0156310)

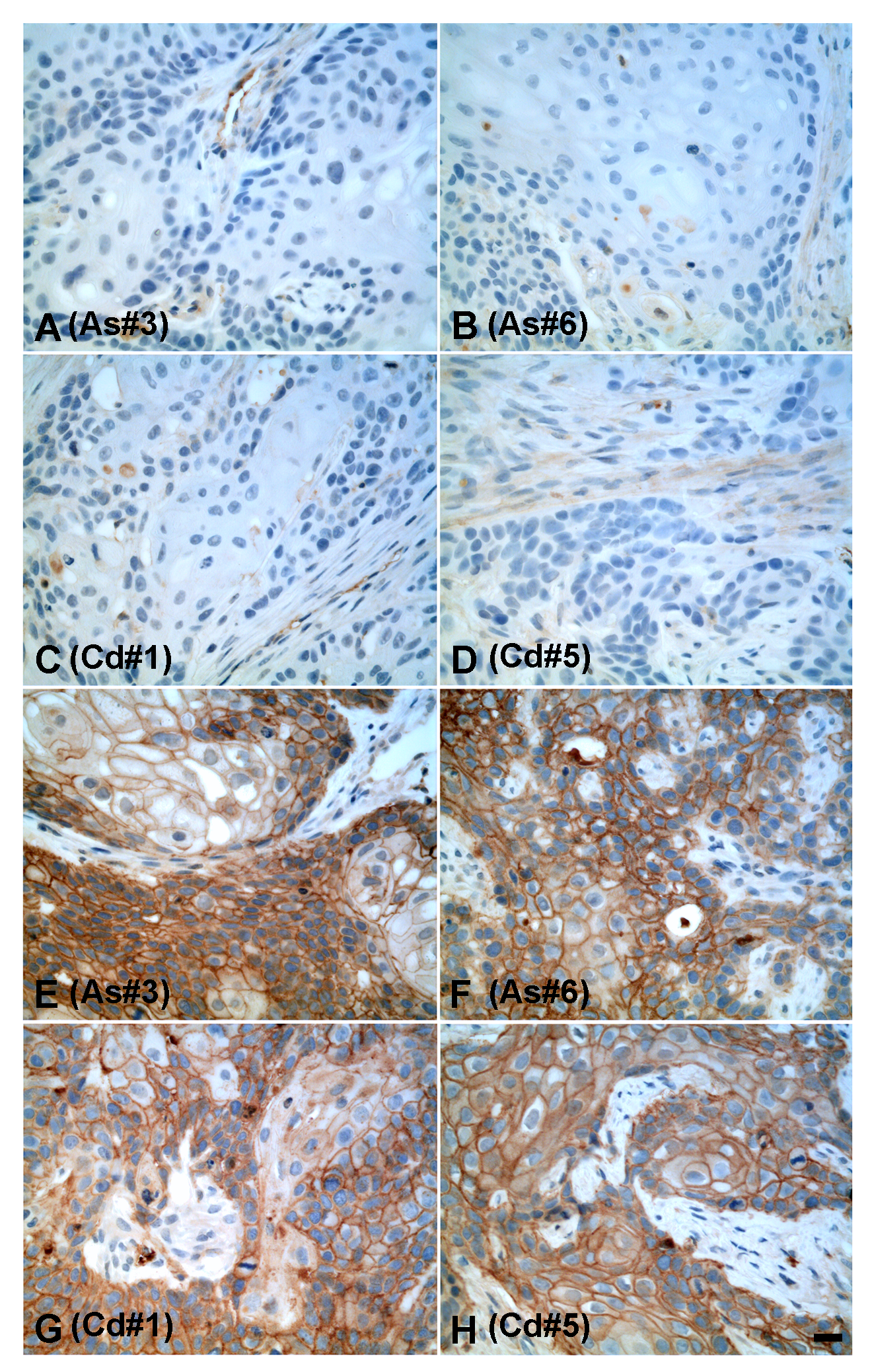

Supplement: S1 Fig — (A-D). Expression of N-cadherin protein in tumor transplants generated from As#3, As#6, Cd#1 and Cd#5 cell lines respectively. (E-H) Expression of E-cadherin protein in tumor transplants generated from As#3, As#6, Cd#1 and Cd#5 cell lines respectively. The brown color indicates the presence of the protein whereas the blue color indicates the nuclei that were stained with the counterstain hematoxylin. All images are at a magnification of X400. Scale bar = 50 μm and is shown for panels A-H. (TIF) [file pone.0156310.s001.tif]

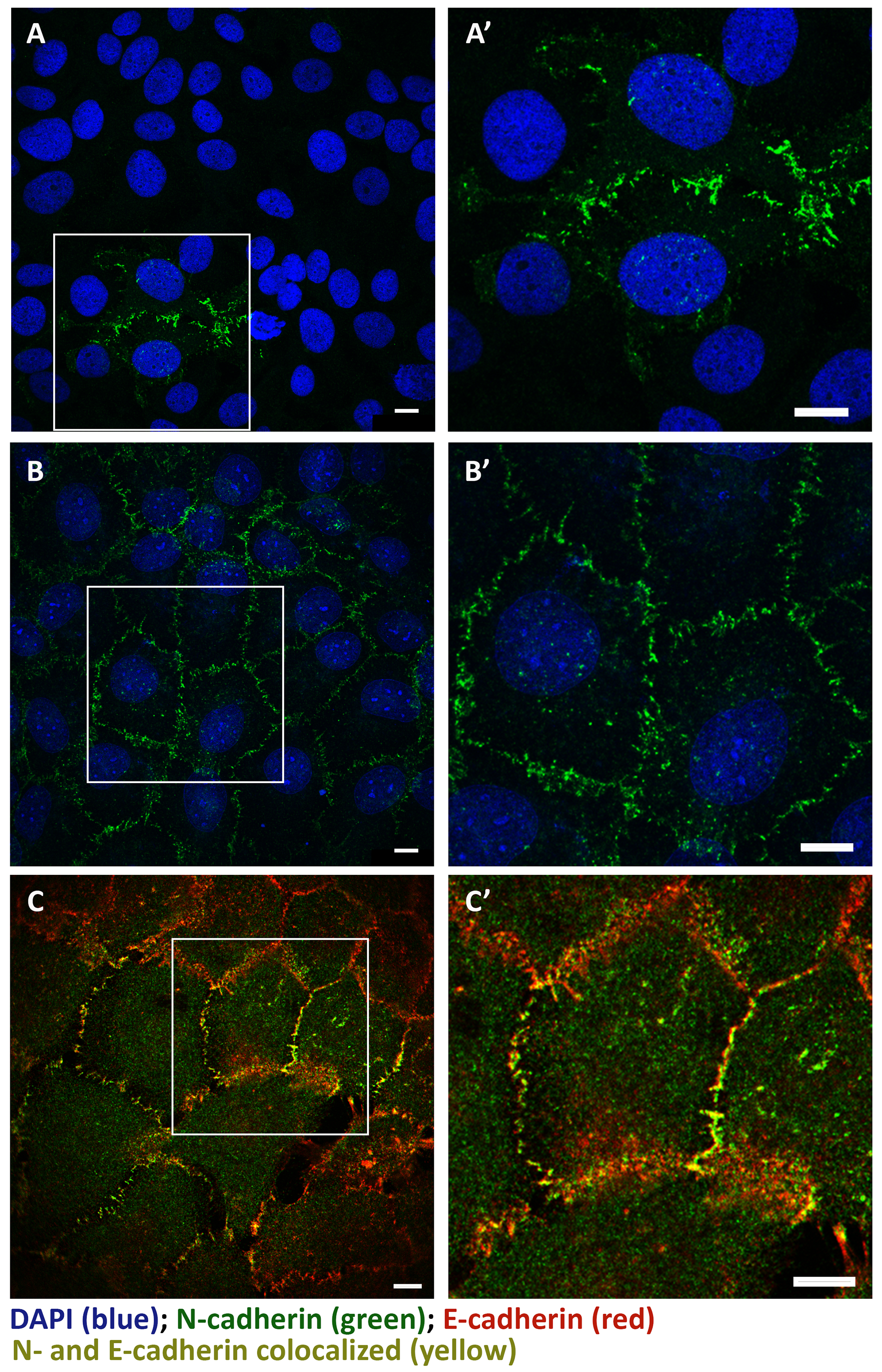

Supplement: S2 Fig — N-cadherin (green) was found to be localized to the plasma membrane, when present, in the parent and transformed UROtsa cell line: (A, A’) UROtsa parent and (B, B’) As#6. Co-localization (yellow) of N-cadherin (green) and E-cadherin (red) is also shown (C, C’). High magnifications of the selected pictures are shown in the panels to the right (A’, B’, C’). The regions magnified are indicated by the white boxes on the lower magnification images. All scale bars = 10 μm. (TIF) [file pone.0156310.s002.tif]

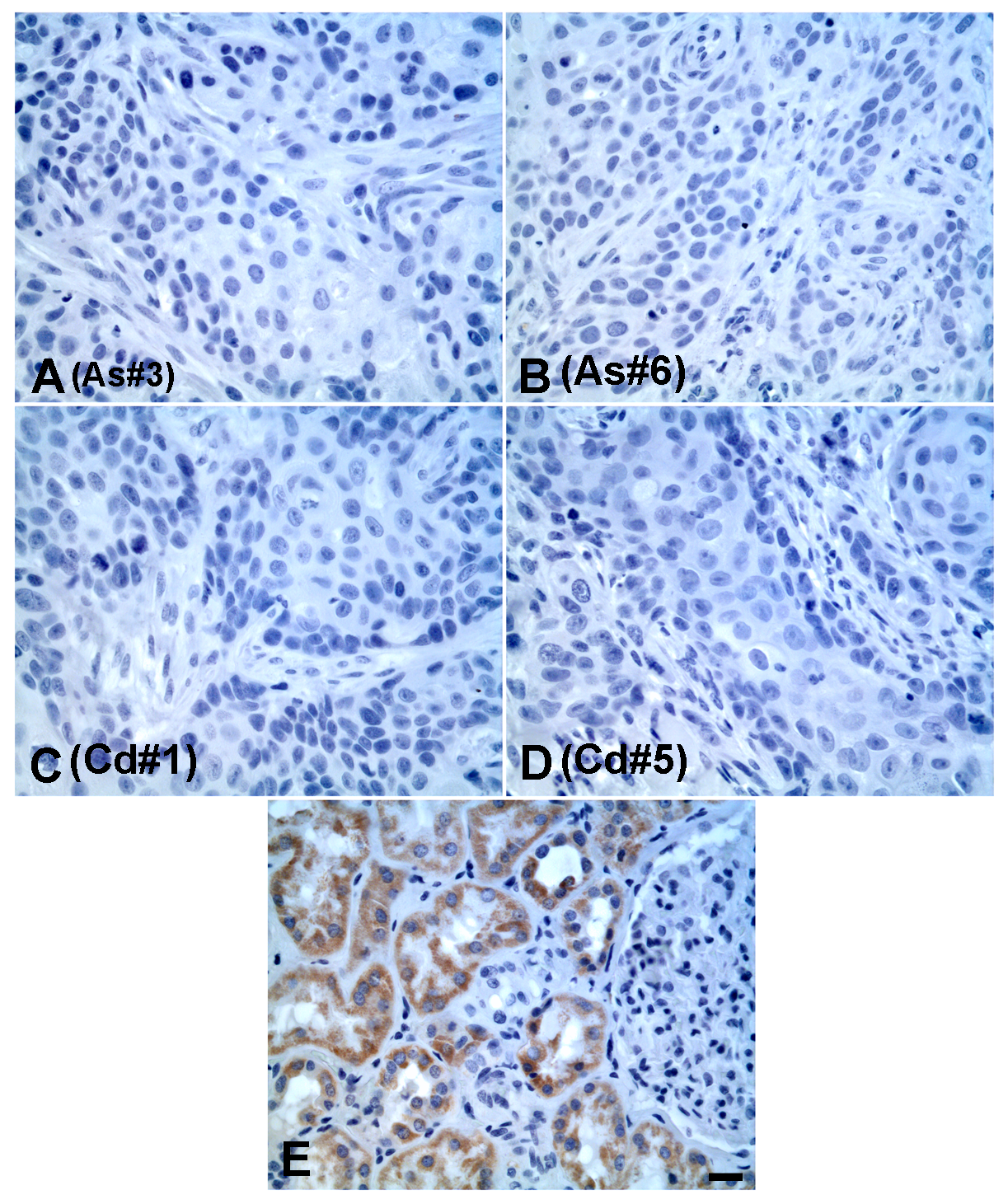

Supplement: S3 Fig — (A-D). Expression of N-cadherin protein in tumor transplants generated from As#3, As#6, Cd#1 and Cd#5 cancer initiating cell spheroids respectively. (E). Human kidney stained for N-cadherin as a positive control. The brown color indicates the presence of the protein whereas the blue color indicates the nuclei that were stained with the counterstain hematoxylin. All images are at a magnification of X400. Scale bar = 50 μm and is shown for panels A-E. (TIF) [file pone.0156310.s003.tif]

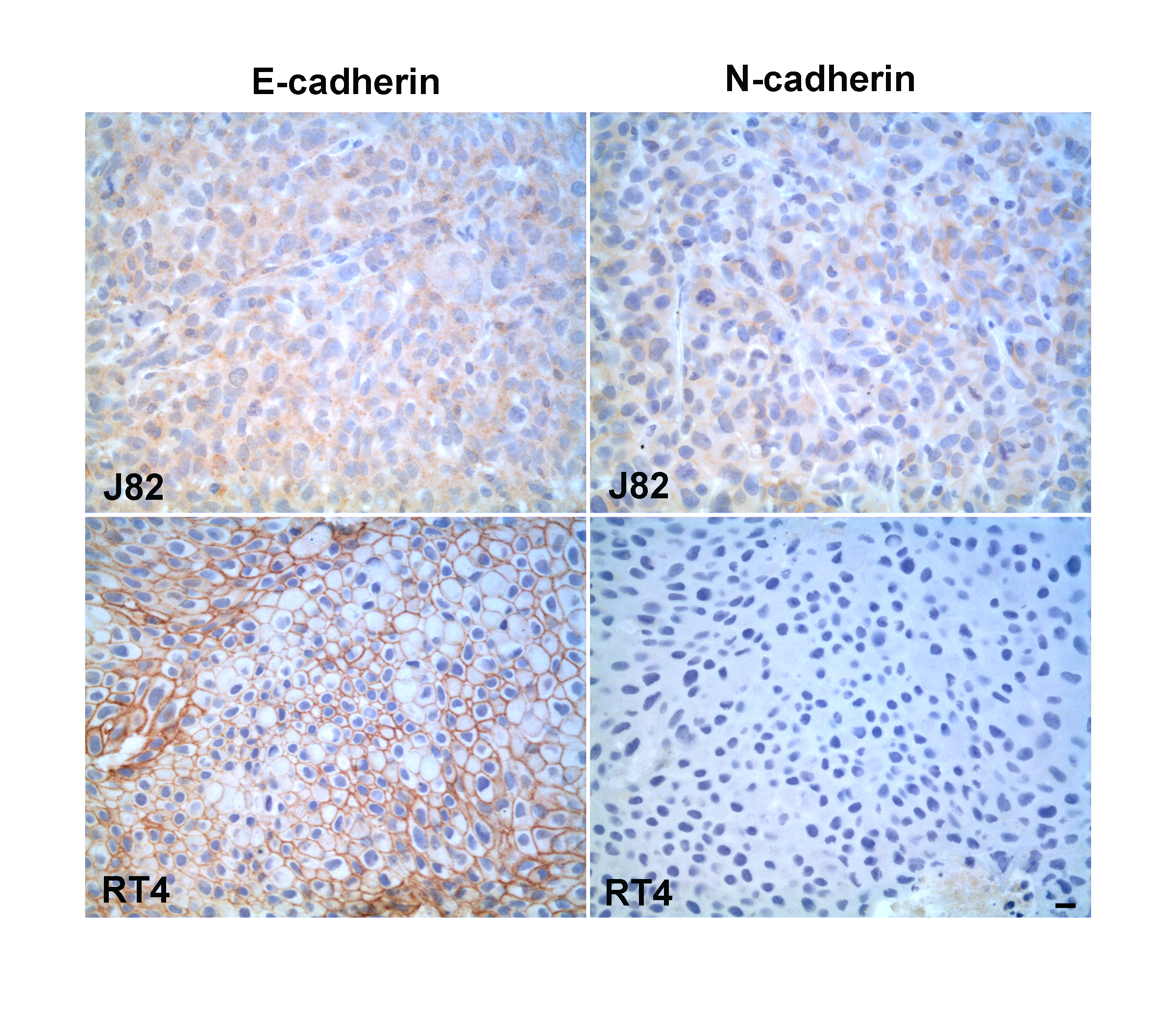

Supplement: S4 Fig — The brown color indicates the presence of the protein whereas the blue color indicates the nuclei that were stained with the counterstain hematoxylin. All images are at a magnification of X400. Scale bar = 50 μm. (TIF) [file pone.0156310.s004.tif]
